# Hypertension burden and associated risk factors among people from the slums in a developing country: evidence from the COMBAT-CVD study

**DOI:** 10.1038/s41371-025-01057-x

**Published:** 2025-08-26

**Authors:** Olumide Ebenezer Olufayo, Osahon Jeffery Asowata, Akinkunmi Paul Okekunle, Onoja Mattthew Akpa

**Affiliations:** 1 Department of Epidemiology and Medical Statistics, Faculty of Public Health, College of Medicine, University of Ibadan, Ibadan, Nigeria; 2 Department of Pharmacology and Toxicology, East Carolina University, Greenville, NC USA; 3 Translational Gerontology Branch, National Institute on Aging, Baltimore, MD USA; 4 Division of Epidemiology, Biostatistics and Environmental Health, School of Public Health, University of Memphis, Memphis, TN USA

**Keywords:** Hypertension, Risk factors

## Abstract

Hypertension remains a public health problem worldwide, particularly in Africa, where the burden is disproportionately high. However, little is known about the burden and factors associated with hypertension among populations living in slums, particularly in Sub-Saharan African countries like Nigeria, where a significant proportion of the population in Africa lives. This study assessed the hypertension burden and risk factors among individuals residing in the slums compared to the overall sample and those from the non-slum areas in Ibadan, Nigeria. In this study, 3635 participants from the door-to-door Community-based Investigation of the Risk Factors for Cardiovascular Diseases study provided information on sociodemographic and lifestyle factors. Blood pressure and anthropometric measurements were carried out using standard procedures. Hypertension was defined as one of the following conditions: systolic blood pressure ≥140 mmHg and diastolic blood pressure ≥90 mmHg, self-reported diagnosis of hypertension by a certified health professional, and current use of anti-hypertensive or blood pressure-lowering medications. Overall, 903 (24.8%) were hypertensive in the entire sample, but 29.4% (170 of 579) of the participants from the slums and 23.9% (733 of 3056) of those living in non-slum areas presented with hypertension. Generally, the odds of hypertension (using “no formal education” as reference) decreased with increasing education in the overall population and those from non-slum areas, with generally suggestive lower odds among those from the slum areas; OR: 0.45; 95% CI: 0.16, 1.25). Lifestyle modification interventions targeting older people who are married and less educated should lessen the burden of hypertension in these slums.

## Background

Hypertension is a major public health problem and a leading risk factor for cardiovascular diseases (CVDs) [1, 2] and disability worldwide [3]. In Sub-Saharan Africa (SSA), evidence suggests that about 11.9–51.7% of Africans have blood pressure scores ≥140/90 mmHg [4]. Demographic transition, ageing, urbanization, and poor lifestyle habits account for the high burden of hypertension in SSA [5]. Previous reports have consistently documented the burden of hypertension in urban and rural areas [3], with limited information about people living in slums, particularly among Africans [5–7].

A slum, according to the United Nations, is a region without essential amenities (sanitation, potable water, and electricity, among others), subpar housing, excessive population, unsafe and unsanitary conditions, unsecured tenancy, and social marginalization [8]. Slum settlements are generally characterized by many factors associated with poor living conditions or inadequate access to essentials such as potable water, good sanitation, and equitable housing [9, 10]. Slum communities [dominate the urban landscape in SSA due to rapid urbanization and the need for cheap labour costs associated with the inability to afford the considerably high cost of living in most of the metropolises in SSA [11]. While several studies have characterized the burden and risk factors of hypertension, specifically in urban or rural areas, little is known about this phenomenon among slum dwellers, particularly in SSA countries like Nigeria [12–14]. Similarly, most studies characterizing the magnitude and risk factors of hypertension in the context of urban-rural disparities in SSA [15–21] are often short of evidence on populations in the slums (as they are primarily classified as part of urban areas) despite the unique demography and social marginalization driving health disparities and determinants in the slums [22]. In addition, slums in SSA are currently undergoing a rapid epidemiological transition with accumulative rates of non-communicable and communicable diseases [23]. Whether the burden and risk factors of hypertension differ in the slum population compared to either rural or urban settings is yet to be clearly understood, particularly in Nigeria [14].

Discerning the burden and risk factors of hypertension among slum settlers is critical to designing context-specific advisories, recommendations and interventions that are necessary for the primary prevention and management of hypertension in populations, particularly in Africa [12, 24–26]. Therefore, this study aimed to characterize the burden of hypertension and its associated risk factors among individuals residing in slums in Ibadan, Nigeria.

## Methods

### Data source and sampling strategy

Data for this study was from the first wave of the Community-based Investigation of the Risk Factors for Cardiovascular Diseases in Ibadan and suburbs (COMBAT-CVDs) carried out in Ibadan, Oyo State, Nigeria. The study was approved (AD13/479/2029A) by the Ethics Review Committee of the Ministry of Health, Oyo State, Nigeria. All methods were performed in accordance with the relevant guidelines and regulations. The COMBAT-CVDs is a community-based door-to-door survey designed to investigate CVD risk factors among adults (≥18 years) living in the Ibadan and its suburbs. Briefly, trained interviewers recruited participants from five local government areas across communities, including slums and non-slum areas around the Ibadan, through a multistage sampling strategy. The study protocol, sampling strategy, sample size estimation, and enrollment, among others, have been detailed elsewhere [27]. Consenting adults participated in the study after signing the consent forms and were interviewed using the standardized World Health Organization STEPwise Approach to NCD Risk Factor Surveillance tool [28–30]. Participants reported information on sociodemographic and lifestyle factors, but physical examinations were conducted for blood pressure assessments, and anthropometry measurements per standard protocols. In all, 3635 participants (out of the total 3638 recruited) were included in this study after excluding three participants without information on hypertension status.

### Characterization of slums

A slum, in this study, was defined in accordance with the United Nations characterization as predominantly settlements lacking essential amenities and housing that are excessively populated, characterized by unsanitary living conditions, irregular tenancy, and social marginalization [8]. In addition, there is substantial evidence alluding to the vulnerability of people living in the slums to a higher risk of CVD due to complex interrelated sociodemographic, socioeconomic, behavioural, and lifestyle factors with living conditions compared to those from non–slum settings [22, 31, 32]. Participants were classified as being from the slums if they were recruited from locations previously described as slums in Ibadan, Nigeria, in an earlier study [10]. These locations are densely populated and differ significantly from rural and urban settings as they are primarily characterized by the following conditions in part or whole: poor living conditions with informal housing, poor road network, lack of drainage and inappropriate wastewater disposal, deteriorating housing, inadequate access to potable water, and poor sanitation, among others [9, 10].

### Definition of hypertension (Exposure variable)

Systolic and diastolic blood pressures were measured thrice (within five-minute intervals of rest) using a blood pressure monitor, and the mean of the last two readings was used to determine the blood pressure measurement for each participant. Hypertension was defined as one of the following conditions: systolic blood pressure ≥140 mmHg and/or diastolic blood pressure ≥90 mmHg, self-reported diagnosis of hypertension by a certified health professional, and current use of anti-hypertensive or blood pressure-lowering medications [33, 34].

### Definition of hypertension risk factors

Participants’ age was reported in years and classified as <50 years (young and middle-aged adults) or ≥50 years (late middle-aged adults) [35]. Biological sex was reported as ‘male’ or ‘female’. At the same time, educational status was self-reported and categorized as ‘no education’ (if the participant had no formal education), ‘primary school’ (if the participant completed primary school education), ‘secondary school’ (if the participant completed a high/secondary school education), and ‘tertiary education & above’ (if the participant completed at least a university education). The employment status was self-reported and classified as ‘employed’ or ‘not employed’. Average monthly income was categorized as ‘<minimum wage (<30,000)’ or ‘≥minimum wage (≥30,000)’ based on the national minimum wage in Nigeria [19]. Marital status was defined as married if the participant reported currently being in a civil or common law marriage with a partner otherwise unmarried (i.e. currently not in any form of marital relationship, whether single, widowed, or divorced) [36]. Participants reported their religious beliefs or affiliations as Christianity, Islam, or others (where their religious beliefs were neither Christianity nor Islam), and ethnic background was self-reported by participants and classified as Yoruba and non-Yoruba, given that the Yoruba ethnic group is the most dominant ethnic group in the study location. Physical activity was measured using the WHO’s Global Physical Activity Questionaire (GPAQ) and classified as active if the participant’s Metabolic Equivalent of Task per week was ≥600 and inactive if otherwise [37]. Participants self-reported tobacco use and smoking status were classified as ‘ever smoked’ on the premise that the participants reported current use of cigarettes or smoked at least 100 cigarettes in a lifetime, else ‘never smoked’ [34]. Alcohol consumption patterns were self-reported, and current alcohol use was defined as the current use of any form of alcoholic drink or product in the last 12 months before the study, else ‘no’ [28]. Also, a family history of CVD was defined as self-reported hypertension, diabetes, stroke, obesity, or heart disease in first and second-degree relatives (mother, father, sister or brother, uncle, aunt, or other close relatives) of the participants [36]. Body mass index (BMI) was estimated from weight (in kilograms) and height (in centimetres and converted to metres) measurements, keeping with standard procedure, as weight in kilograms divided by the square of height in metres square, and obesity was classified as BMI ≥ 30 kg/m^2^ [38].

### Statistical analysis

Participants’ characteristics were presented in count (percentage) and mean (±standard deviation-SD) for categorical and continuous variables, respectively. The chi-square test (for categorical variables) and independent sample t-test (for the continuous variables) were used to evaluate the bivariate association between hypertension status and participants’ characteristics in the entire sample and stratified by participants’ residence (slums and non-slum areas). Furthermore, logistic regression models were used to estimate the odds ratio (OR) and 95% confidence interval (CI) for hypertension in the entire sample, stratified by participants’ residence and age groups (<50 years vs ≥50 years) [35], to determine context-specific factors associated with hypertension in this population. Variables were included in the model if statistically significant (*P* < 0.05) in the bivariate analysis. All statistical analyses were carried out using Statistical Package for Social Sciences (SPSS Inc., Chicago, IL, USA) version 25.0 at a two-sided *P* < 0.05.

## Results

### Characteristics of study participants

Overall (Table 1), 3635 participants with a mean age of 35.33 ± 15.2 years (579; 15.9% were from slum areas) were included in this study. Also, 1982 (54.5%) were males, 2893 (79.6%) were employed, 2162 (59.5%) were married, and 2200 (60.5%) earned less than N30,000 (Table 1). Also, 685 (18.8%) were physically inactive, 442 (12.2%) had ever used tobacco, 2623 (72.2%) currently use alcohol, 435 (12.0%) were obese, and 535 (14.7%) reported a family history of CVD. The mean systolic and diastolic blood pressures were 120.5 ± 18.3 and 79.0 ± 12.6 mmHg, respectively.

**Table 1:** Characteristics of study participants categorized by hypertension status in the COMBAT-CVD study.

	All participants				Participants from the slums			Participants from non-slums areas		
Variables	All	Non-hypertensives	Hypertensive	P-value	Non-hypertensives	Hypertensive	P-value	Non-hypertensives	Hypertensive	P-value
	*n* = 3635	*n* = 2732	*n* = 903 (24.8%)		*n* = 409	*n* = 170 (29.4%)		*n* = 2323	*n* = 733 (23.9%)	
Age (mean ± SD), in years	35.33 ± 15.2	31.8 ± 13.8	45.1 ± 14.3	<0.001	35.6 ± 20.1	44.4 ± 12.4	0.254	31.2 ± 12.2	46.3 ± 14.7	<0.001
<50	2965 (59.0)	2432 (89.0)	533 (59.0)	<0.001	340 (83.1)	107 (62.9)	<0.001	2092 (90.1)	426 (58.1)	<0.001
≥50	670 (18.4)	300 (11.0)	370 (41.0)		69 (16.9)	63 (37.1)		231 (9.9)	307 (41.9)	
Sex										
Female	1653 (45.5)	1248 (45.7)	405 (44.9)	0.66	143 (35.0)	57 (33.5)	0.74	1105 (47.6)	348 (47.5)	0.97
Male	1982 (54.5)	1484 (54.3)	498 (55.1)		266 (65.0)	113 (66.5)		1218 (52.4)	385 (52.5)	
Educational Status										
None	114 (3.1)	54 (2.0)	60 (6.6)	<0.001	8 (2.0)	11 (6.5)	0.001	46 (2.0)	49 (6.7)	<0.001
Primary	517 (14.2)	304 (11.1)	213 (23.6)		72 (17.6)	46 (27.1)		232 (10.0)	167 (22.8)	
Secondary	1933 (53.2)	1507 (55.2)	426 (47.2)		244 (59.6)	90 (52.9)		1263 (54.3)	336 (45.8)	
Tertiary	1071 (29.5)	867 (31.7)	204 (22.6)		85 (20.8)	23 (13.5)		782 (33.7)	181 (24.7)	
Employment Status										
Unemployed	742 (20.4)	654 (23.9)	88 (9.7)	<0.001	66 (16.1)	6 (3.5)	<0.001	588 (25.3)	82 (11.2)	<0.001
Employed	2893 (79.6)	2078 (76.1)	815 (90.3)		343 (83.9)	164 (96.5)		1735 (74.7)	651 (88.8)	
Average monthly income										
<30,000	2200 (60.5)	1710 (62.6)	490 (54.3)	<0.001	221 (54.0)	70 (41.2)	0.01	1489 (64.1)	420 (57.3)	0.001
≥30,000	1435 (39.5)	1022 (37.4)	413 (45.7)		188 (46.0)	100 (58.8)		834 (35.9)	313 (42.7)	
Marital status										
Unmarried	1473 (40.5)	1349 (49.4)	124 (13.7)	<0.001	179 (43.8)	28 (16.5)	<0.001	1170 (50.4)	96 (13.1)	<0.001
Married	2162 (59.5)	1383 (50.6)	779 (86.3)		230 (56.2)	142 (83.5)		1153 (49.6)	637 (86.9)	
Religion										
Christianity	1916 (52.7)	1454 (53.2)	462 (51.2)	0.40	193 (47.2)	73 (42.9)		1261 (54.3)	389 (53.1)	0.57
Islam	1698 (46.7)	1264 (46.3)	434 (48.1)		214 (52.3)	96 (56.5)	0.64	1050 (45.2)	338 (46.1)	
Others	21 (0.6)	14 (0.5)	7 (0.8)		2 (0.5)	1 (0.6)		12 (0.5)	6 (0.8)	
Ethnicity										
Yoruba	3349 (92.1)	2516 (92.1)	833 (92.2)	0.88	377 (92.2)	158 (92.9)	0.75	2139 (92.1)	675 (92.1)	0.99
Non -Yoruba	286 (7.9)	216 (7.9)	70 (7.8)		32 (7.8)	12 (7.1)		184 (7.9)	58 (7.9)	
Physical activity										
Inactive	685 (18.8)	505 (18.5)	180 (19.9)	0.33	39 (9.5)	25 (14.7)	0.07	466 (20.1)	155 (21.1)	0.5274
Active	2950 (81.2)	2227 (81.5)	723 (80.1)		370 (90.5)	145 (85.3)		1857 (79.9)	578 (78.9)	
Ever used Tobacco use.										
No	3193 (87.8)	2432 (89.0)	761 (84.3)	<0.001	334 (81.7)	125 (73.5)	0.03	2098 (90.3)	636 (86.8)	0.006
Yes	442 (12.2)	300 (11.0)	142 (15.7)		75 (18.3)	45 (26.5)		225 (9.7)	97 (13.2)	
Current Alcohol use										
No	1012 (27.8)	761 (27.9)	251 (27.8)	0.97	107 (26.2)	45 (26.5)	0.94	654 (28.2)	206 (28.1)	0.98
Yes	2623 (72.2)	1971 (72.1)	652 (72.2)		302 (73.8)	125 (73.5)		1669 (71.8)	527 (71.9)	
Family history of CVD										
No	3100 (85.3)	2358 (86.3)	742 (82.2)		360 (88.0)	146 (85.9)	0.48	1998 (86.0)	596 (81.3)	0.002
Yes	535(14.7)	374 (13.7)	161 (17.8)		49 (12.0)	24 (14.1)		325 (14.0)	1373 (18.7)	
Body Mass Index										
Nonobese(<30 kg/m^2^)	3200(88.0)	2501 (91.5)	699 (77.4)	<0.001	366 (89.5)	141 (82.3)	0.03	2135 (91.9)	558 (76.1)	<0.001
Obese(≥30 kg/m^2^)	435 (12.0)	231 (8.5)	204 (22.6)		43 (10.5)	29 (17.1)		188 (8.1)	175 (23.9)	
Systolic blood pressure (Mean ± SD)	120.5 ± 18.3	113.6 ± 11.0	141.1 ± 20.5	<0.001	115.5 ± 10.8	1380 ± 20.6	<0.001	113.3 ± 11.0	141.9 ± 20.4	<0.0001
Diastolic blood pressure (Mean ± SD)	79.0 ± 12.6	74.2 ± 7.9	93.6 ± 13.0	<0.001	75.8 ± 7.8	91.6 ± 13.4	<0.001	73.9 ± 79	94.0 ± 12.9	<0.0001

### Prevalence of hypertension

Overall, 903 (24.8%) participants had hypertension in the entire sample (Table 1), but more males, 498 (55.1%), presented with hypertension than females, 405 (44.9%). The preponderance of hypertension was likely among those < 50 years 533 (59.0%), having secondary school education 426 (47.2%), being employed 815 (90.3%), being married 779 (86.3%), earning <30,000 naira monthly 490 (54.3%), tobacco smoking 142 (15.7%), and among those with obesity 204 (22.6%). Hypertension prevalence among participants from the slums was 170 (29.4%), the preponderance of hypertension was probable among those <50 years 107 (62.9%), being employed 164 (96.5%), and married 142 (83.5%), with generally high proportions but not among those earning < 30,000 naira monthly 70 (41.2%) and tobacco smokers 45 (26.5%). The prevalence of hypertension among those from the non-slums was 733 (23.9%), and the findings on the preponderance of hypertension generally mirrored findings in the overall sample.

### Factors associated with hypertension

**Fig. 1:**
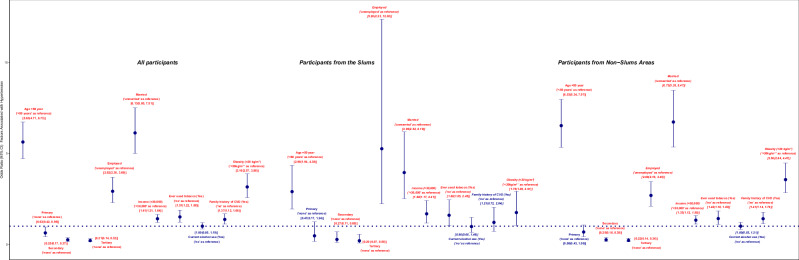
Forest plot describing the crude odds of the association of sociodemographic, lifestyle, anthropometric, and clinical characteristics with hypertension. The crude odds ratios (95% confidence intervals) of factors associated with hypertension were presented for all participants, those from the slums, and those from non-slum areas.

**Fig. 2:**
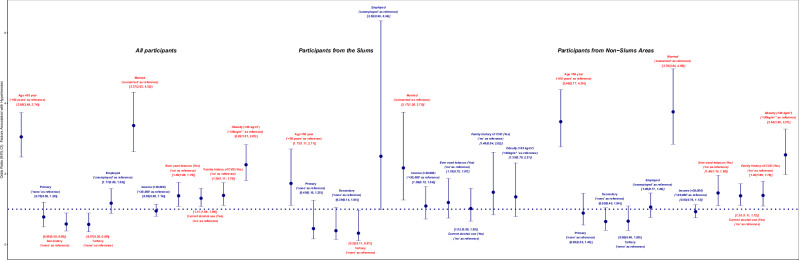
Forest plot describing the adjusted odds of the association of sociodemographic, lifestyle, anthropometric, and clinical characteristics with hypertension in a single regression model. The adjusted odds ratios (95% confidence intervals) of factors associated with hypertension were presented for all participants, those from the slums, and those from non-slum areas.

Factors with statistically significant *P* values in the bivariate associations were included in the regression models, and the crude OR and 95% CI of the associations are presented in Fig. 1 and Table 2. When the factors were included in single regression model (Fig. 2 and Table 3), the adjusted OR of hypertension among those ≥50 years (compared to those < 50 years) was (OR: 3.05; 95%CI: 2.48, 3.74) in the overall sample, higher (OR: 3.48; 95%CI: 2.77, 4.39) among those from non-slum areas, but lower (OR: 1.73; 95%CI: 1.11, 2.71) among participants from the slum. Generally, the odds of hypertension (using no formal education as reference) suggestively decreased (though not statistically significant for primary and secondary education) with increasing education in the overall population and those from non-slum areas with generally lower odds among those from the slum areas; OR: 0.45; 95%CI: 0.16, 1.25) – for primary education, OR: 0.39; 95%CI: 0.14, 1.05 – for secondary education and OR: 0.32; 95%CI: 0.11, 0.97 – for those with tertiary education in the slums. Among those employed (with unemployed as a reference, the odds of hypertension were OR: 1.17; 95%CI: 0.88, 1.58 and OR: 1.06; 95%CI: 0.77, 1.46 in the overall sample and among those from non-slum areas, but higher among employed participants from the slums; OR: 2.50; 95%CI: 0.99, 6.34. However, the odds of hypertension among married participants in comparison with those unmarried were higher in the overall population (OR: 3.37; 95%CI: 2.63, 4.32) and among those from non-slum areas (OR: 3.76; 95%CI: 2.84, 4.98), but lower among married participants from the slums (OR: 2.17; 95%CI: 1.26, 3.75). Having a higher income of 30,00 naira or more was associated with lower odds of hypertension in the overall sample (OR: 0.95; 95%CI: 0.80, 1.14) and among non-slum dwellers (OR: 0.93; 95%CI: 0.76, 1.13), but not among those living in the slums (OR: 1.09; 95%CI: 0.72, 1.64). Tobacco smoking, current alcohol use and having a family history of CVD presented relatively comparable deleterious odds with hypertension independent of the residential status, but the odds of hypertension among obese participants (compared to non-obese participants) appear lower among those from the slums (OR: 1.35; 95%CI: 0.79, 2.31), but higher in the overall sample (OR: 2.26; 95%CI: 1.81, 2.83) and among those from non-slum areas (OR: 2.54; 95%CI: 1.98, 3.27).

**Table 2:** The crude odds of the association of sociodemographic, lifestyle, anthropometric, and clinical characteristics with hypertension.

		Stratified by Residence		Stratified by Age	
	All participants	Participants from the Slums	Participants from Non-SlumsAreas	<50 years	≥50 years
	OR (95% CI)	OR (95% CI)	OR (95% CI)	OR (95% CI)	OR (95% CI)
Age groups					
<50	1	1	1		
≥50	5.63 (4.71, 6.73)	2.90 (1.94, 4.35)	6.53 (5.34, 7.97)		
Educational Status					
None	1	1	1	1	1
Primary	0.63 (0.42, 0.95)	0.47 (0.17, 1.24)	0.68 (0.43, 1.06)	1.29 (0.58, 2.87)	0.64 (0.38, 1.09)
Secondary	0.25 (0.17, 0.37)	0.27 (0.11, 0.69)	0.25 (0.16, 0.38)	0.56 (0.26, 1.22)	0.70 (0.42, 1.17)
Tertiary	0.21 (0.14, 0.32)	0.20 (0.07, 0.55)	0.22 (0.14, 0.34)	0.51 (0.24, 1.12)	0.66 (0.36, 1.20)
Employment Status					
Unemployed	1	1	1	1	1
Employed	2.92 (2.30, 3.69)	5.26 (2.23, 12.38)	2.69 (2.10, 3.45)	3.53 (2.59, 4.80)	0.75 (0.44, 1.27)
Marital status					
Unmarried	1	1	1	1	1
Married	6.13 (5.00, 7.51)	3.95 (2.52, 6.19)	6.73 (5.35, 8.47)	4.17 (3.35, 5.18)	2.49 (0.45, 13.67)
Average monthly income					
<30,000	1	1	1	1	1
≥30,000	1.41 (1.21, 1.64)	1.68 (1.17, 2.41)	1.33 (1.12, 1.58)	1.61 (1.33, 1.94)	0.63 (0.47, 0.89)
Ever used tobacco					
No	1	1	1	1	1
Yes	1.51 (1.22, 1.88)	1.60 (1.05, 2.45)	1.42 (1.10, 1.83)	1.48 (1.13, 1.94)	1.19 (0.79, 1.82)
Current alcohol use					
No	1	1	1	1	1
Yes	1.00 (0.85, 1.19)	0.98 (0.66, 1.48)	1.00 (0.83, 1.21)	1.17 (0.94, 1.46)	1.12 (0.81, 1.54)
Family history of CVD					
No	1	1	1	1	1
Yes	1.37 (1.12, 1.68)	1.21 (0.72, 2.04)	1.41 (1.14, 1.76)	1.42 (1.11, 1.82)	1.92 (1.28, 2.87)
Body Mass Index					
Non-obese (<30 kg/m^2^)	1	1	1	1	1
Obese(≥30 kg/m^2^)	3.16 (2.57, 3.88)	1.75 (1.05, 2.91)	3.56 (2.84, 4.47)	3.30 (2.56, 4.26)	1.22 (0.80, 1.85)

**Table 3:** Independent association of sociodemographic, lifestyle, anthropometric, and clinical characteristics with hypertension in a single regression model.

		Stratified by Residence		Stratified by Age	
	All participants	Participants from the Slums	Participants from Non-Slums Areas	<50 years	≥50 years
	OR (95% CI)	OR (95% CI)	OR (95% CI)	OR (95% CI)	OR (95% CI)
Age groups					
<50	1	1	1		
≥50	3.05 (2.48, 3.74)	1.73 (1.11, 2.71)	3.48 (2.77, 4.39)		
Educational Status					
None	1	1	1	1	1
Primary	0.78 (0.50, 1.20)	0.45 (0.16, 1.25)	0.89 (0.55, 1.45)	1.25 (0.55, 2.89)	0.63 (0.37, 1.08)
Secondary	0.58 (0.38, 0.89)	0.39 (0.14, 1.05)	0.65 (0.40, 1.04)	0.75 (0.34, 1.68)	0.68 (0.39, 1.19)
Tertiary	0.57 (0.36, 0.89)	0.32 (0.11, 0.97)	0.66 (0.40, 1.09)	0.72 (0.32, 1.63)	0.65 (0.34, 1.24)
Employment Status					
Unemployed	1	1	1	1	1
Employed	1.17 (0.88, 1.58)	2.50 (0.99, 6.34)	1.06 (0.77, 1.46)	1.42 (0.99, 2.04)	0.76 (0.44, 1.32)
Marital status					
Unmarried	1	1	1	1	1
Married	3.37 (2.63, 4.32)	2.17 (1.26, 3.75)	3.76 (2.84, 4.98)	2.96 (2.29, 3.83)	2.16 (0.38, 12.39)
Average monthly income					
<30,000	1	1	1	1	1
≥30,000	0.95 (0.80, 1.14)	1.09 (0.72, 1.64)	0.93 (0.76, 1.13)	1.14 (0.92, 1.41)	0.62 (0.44, 0.86)
Ever used tobacco					
No	1	1	1	1	1
Yes	1.38 (1.08, 1.76)	1.19 (0.75, 1.87)	1.46 (1.10, 1.96)	1.36 (1.02, 1.83)	1.38 (0.89, 2.14)
Current alcohol use					
No	1	1	1	1	1
Yes	1.31 (1.08, 1.59)	1.02 (0.66, 1.59)	1.38 (1.11, 1.72)	1.30 (1.03, 1.65)	1.28 (0.91, 1.82)
Family history of CVD					
No	1	1	1	1	1
Yes	1.39 (1.11, 1.75)	1.48 (0.84, 2.62)	1.39 (1.09, 1.79)	1.50 (1.15, 1.95)	1.17 (0.76, 1.79)
Body Mass Index					
Non-obese (<30 kg/m^2^)	1	1	1	1	1
Obese(≥30 kg/m^2^)	2.26 (1.81, 2.83)	1.35 (0.79, 2.31)	2.54 (1.98, 3.27)	2.39 (1.83, 3.13)	2.05 (1.36, 3.11)

Furthermore, the distribution of hypertension odds varied widely upon stratification by age groups (<50 vs ≥50 years) – Table 3. Generally, being married, tobacco smoking, current alcohol use, having a family history of CVD and being obese were associated with higher odds of hypertension, but education was inversely related to the prevalence of hypertension, independent of age groups. However, being employed (compared to those unemployed) was directly and inversely related to hypertension prevalence among those <50 years (OR: 1.42; 95%CI: 0.99, 2.04) and ≥50 years (OR: 0.76; 95%CI: 0.44, 1.32), respectively. Similarly, earning 30,000 naira or more (compared with those who earn <30,000 naira) was directly and inversely related to hypertension prevalence among those <50 years (OR: 1.14; 95%CI: 0.92, 1.41) and ≥50 years (OR: 0.62; 95%CI: 0.44, 0.86), respectively.

## Discussion

In this study, we characterized the burden of hypertension and its context-specific associated factors among participants from the slums compared to the overall sample and those from non-slum areas in Ibadan, Nigeria. The dynamics of hypertension burden and risk factors appear similar, regardless of residence status, but largely discordant in terms of magnitude, especially among those residing in slums. The prevalence of hypertension was relatively higher among participants from the slums compared to the non-slum and the entire sample. Precisely, the distribution of hypertension odds among late middle-aged adults (≥50 years) was lower among those from the slums but higher in non-slum areas. Also, Having a higher education was generally associated with lower odds of hypertension in this population, especially among those from the slums. Being employed was generally associated with higher odds of hypertension, but the odds were more substantial among employed participants from the slums. The odds of hypertension were lower among higher-income earners from non-slum areas, contrary to the higher odds observed among similar populations in the slums.

The prevalence of hypertension was higher among those from the slums than among the overall population under study and those from non-slum areas. This study’s findings mirror that which was reported in slum settlements in Bangladesh – 28.3% [39], India – 17.4% [40], and Argentina – 36.1% [41], among others. Several reasons account for the hugely disproportionate high hypertension prevalence, ranging from exposure to multiple lifestyle risk factors of hypertension to poor blood pressure management [42], especially among those living in slums, where access to health services has been hindered by multiple barriers attributable to disproportionate lack of basic essential municipal services [43]. In tandem with our findings, modest evidence in slums from other climes suggests evolving epidemiological transitions to higher CVD risk arising from multifaceted interaction of living conditions, lifestyle and access to treatment and healthcare in the slums [22, 31, 32]. People in the slums often live in poverty with limited housing and critical necessities, accompanied by overcrowding and lack of access to potable water, which could induce chronic stress and health risks that could increase the burden of hypertension in this setting [22]. Also, health information is inadequate, given that healthcare is underutlized or largely sourced from out-of-pocket, thereby discouraging health-seeking behaviour that could inform early diagnosis and prompt management of hypertension in this setting [44, 45]. Similarly, the limited pecuniary opportunities and job instability, with unsafe living conditions, could promote chronic stress and contribute to the high hypertension burden in the slums [46]. In the same vein, education is a critical driver for most health outcomes, as with hypertension in this study, given that education improves the odds of engaging in healthy lifestyle changes by making rational decisions to improve quality of life. We observed that hypertension odd appeared lower among those with higher education in the slums. In tandem with our findings, hypertension prevalence has been reported to be higher among those with lower educational status [47]. Similarly, a statistically significant inverse relationship was observed between hypertension prevalence and higher educational status [48]. The findings from these previous studies correspond to our observations in the present study. This is because education plays a vital role in increasing awareness, self-care practices, management, and treatment of hypertension.

The case for the more substantial odds of hypertension among employed participants from the slum speaks to the pressure imposed by the demand of seeking income to meet needs for sustaining life, which might be aggravated by the poor living conditions experienced in the slums. Also, we observed that the odds of hypertension were higher with higher income among those from the slums but lower among those from non-slum areas. Our findings were supported by a similar study in a sample of participants from low-income neighbourhoods from 12 countries, revealing that high-income earners were 8% more likely to be hypertensive [49], thereby buttressing that aside from the increase in hypertension burden, there is a high tendency for the undiagnosed burden of hypertension among the slum inhabitants due to a lack of awareness of health screening and medical check-ups [50]. Also, the implications of unhealthy lifestyle practices such as smoking, alcohol use, and excessive weight gain, among others, in aggravated hypertension odds independent of residence status are in tandem with earlier reports [29]. Previous studies revealed that socioeconomic factors such as employment status and income play a significant impact as risk factors for hypertension, which can also promote increased hypertension risk among slum dwellers [49, 51].

Furthermore, demographic and lifestyle characteristics were generally associated with higher odds of hypertension, except for education, which was independent of age groups. Socioeconomic factors, including employment status and income, exhibited a divergent relationship with hypertension prevalence—employed young and middle-aged adults with higher earning power presented with higher odds of hypertension, while late middle-aged adults with employment and higher earnings presented with lower odds of hypertension. Also, the present study found that excessive weight gain is significantly related to hypertension for slum dwellers. This corresponds to a previous study that highlighted that excessive weight gain could lead to obesity and hypertension [52].

This study’s limitations include the inability to draw a causal conclusion, the likelihood of bias concerning selection, residual confounding, and statistical power to detect differences in the odds. Information bias cannot be exclusively ruled out, especially for self-reported information. However, participants provide factually accurate responses in exchange for ensuring anonymity, especially during the consenting process before participation. In addition, trained personnel who conducted the interview adopted sound interviewing methods to clarify abstract perceptions/impressions in questions. In some cases, trained personnel triangulated responses by asking the same question in multiple ways for additional or confirmatory supporting information to assert the reliability of their responses. Our study has potential strengths, including being one of the earliest community-based door-to-door surveys investigating hypertension and its associated risk factors among those living in the slums in this setting. It also provides evidence-based information to guide appropriate context-specific multimodal interventions for the primordial prevention of the already high burden of hypertension in an indigenous African population.

## Conclusion

The burden of hypertension and associated risk factors appears to be worse among those residing in the slums. Targeted interventions with a key interest in improving education and health literacy to promote lifestyle modification and improved health-seeking behaviour with adequate access to intervention/tools for blood pressure management would be promising in reducing the burden of hypertension in this setting. Relevant stakeholders, including governments, non-governmental agencies and donor agencies, should consider extending basic social amenities and promoting opportunities to empower and improve quality of life of the populations in the slums.

## Summary

### What is known about the topic?

- Health inequities driving the hypertension burden in Africa keep evolving, and little is known about the burden and factors associated with hypertension among those living in slums, particularly in Sub-Saharan African countries like Nigeria, where a significant proportion of the population in Africa lives.

### What this study adds?

- Approximately three in every ten persons living in the slum presented with hypertension, and the odds of having hypertension reduced with increasing level of formal education.
- Our findings indicated the need for improved health-seeking behaviour, with adequate access to appropriate information/tools for blood pressure management, would be promising in promoting relevant lifestyle modification and intervention targeted at reducing the burden of hypertension in this setting.

## Data Availability

The participant’s data from which the results presented in this article (text, tables, and figures) have been de-identified. The dataset is available upon reasonable request, and a proposal to access the data should be directed to the COMBAT-CVDs study data access committee (PI: onojamatthew@yahoo.co.uk). Data requestors will need to sign a data access agreement.
